# Transient and selective effects of acute exercise intensity on response inhibition: an EEG study

**DOI:** 10.3389/fnhum.2026.1706674

**Published:** 2026-02-19

**Authors:** Masaki Takayose, Ryo Koshizawa, Kazuma Oki, Christina Thunberg, René J. Huster

**Affiliations:** 1 Department of Liberal Arts and Basic Sciences, College of Industrial Technology, Nihon University, Chiba, Japan; 2 Multimodal Imaging and Cognitive Control Lab, Department of Psychology, University of Oslo, Oslo, Norway; 3 College of Economics, Nihon University, Tokyo, Japan; 4 College of Science and Technology, Nihon University, Chiba, Japan

**Keywords:** acute exercise, cognitive control, event-related potentials, exercise intensity, response inhibition, stop-signal task

## Abstract

Acute aerobic exercise can transiently influence cognitive control, but how exercise intensity and recovery timing shape response inhibition and its neural correlates remains insufficiently understood. This EEG study tested how different exercise intensities modulate response inhibition, behavioral performance, and event-related potentials (ERPs) across pre-exercise, during exercise, and two recovery phases. Twenty-one healthy young adults (6 females, 15 males) participated and self-selected an exercise-intensity level at registration, exercising at a low, moderate or high intensity based on heart rate reserve. Participants performed a stop-signal task (SST) in four experimental conditions: pre-exercise (control), during exercise (exercise), immediately post-exercise (recovery_1), and after heart rate had returned to near resting levels (recovery_2). Behavioral performance indices, including reaction times and accuracy measures, and ERP components (P2, N2, and P3) were assessed. Behavioral analyses revealed significantly reduced reaction times during the exercise condition compared to the control condition, particularly in the high-intensity exercise group. These improvements were transient, with performance returning to baseline or slowing during the recovery phases. ERP analyses showed selective, phase-dependent modulation. Specifically, the N2 amplitude during go trials was significantly reduced during exercise, indicating altered engagement of go-related control processes rather than uniquely implying improved efficiency, while the N2 amplitude during stop trials remained unchanged. Additionally, the P3 amplitude during unsuccessful stop trials showed a modest increase in the immediate post-exercise recovery period, suggesting a transient modulation of evaluation/monitoring processes. Overall, these findings indicate phase-specific exercise effects in a response inhibition task, with facilitation of response execution and stopping during exercise flanked by recovery-phase ERP modulations. By systematically characterizing performance and ERPs across control, exercise, and recovery periods with EEG recorded during exercise with different intensities, we found that in-exercise behavioral gains were most pronounced at higher intensities, whereas persistence after exercise was limited. Overall, acute exercise temporarily enhances response execution and stopping efficiency during exercise—especially at higher intensities—but these effects do not appear to continue into short post-exercise recovery windows in the present protocol.

## 1 Introduction

It is well known that habitual exercise improves cognitive functions such as executive function and memory in humans. A systematic review and meta-analysis by Ludyga et al. (2020) have reported that exercise is associated with cognitive benefits across populations, although the magnitude of these effects can vary as a function of exercise characteristics (e.g., modality and dose) and individual factors. The cognitive effects of long-term exercise habits have been linked to multiple neurobiological pathways, including structural and functional brain changes as well as altered neurotrophic and vascular processes (Ferrer-Uris et al., 2022).

In recent years, there has been an increase in studies examining the effects of acute exercise on cognitive functioning. These studies are crucial for developing interventions to enhance cognitive performance in specific situations and for unraveling mechanisms underlying changes in brain function following habitual exercise. Some studies point toward immediate benefits of exercise on cognitive processing (e.g., Patrick et al., 2025). However, acute exercise does not always facilitate cognition. For example, during high-intensity aerobic exercise, visual sensory processing showed a transient improvement that rapidly returned to baseline while a demanding cognitive task showed no exercise-related change (Lambourne et al., 2010). Other work suggests that exercise may prevent performance decline under some conditions rather than producing broad improvements (Coles and Tomporowski, 2008). A systematic review and meta-analysis further suggested that acute exercise effects on executive functions are statistically significant but typically small, and that performance gains are more likely when baseline performance is low and/or task demands are high (Ishihara et al., 2021). These observations imply that acute exercise effects may be selective and context-dependent, and that moderators such as exercise intensity and the timing of assessment relative to exercise need to be carefully considered.

Inhibitory control, working memory, and cognitive flexibility are considered to be core components of executive functions (Diamond, 2013). Inhibitory control can be further divided into response inhibition, which suppresses an already prepared response (Logan and Cowan, 1984), and cognitive inhibition, which attenuates unnecessary information that distracts attention (MacLeod, 2007). Recent neurophysiological studies suggest that acute exercise can modulate cognitive inhibition, but the direction and magnitude of effects appear to depend on exercise intensity and task demands. For example, Stroop-based fNIRS studies reported greater post-exercise prefrontal activation after low-to-moderate cycling (Yanagisawa et al., 2010; Byun et al., 2014), whereas during high-intensity exercise, accuracy decreased under high cognitive load while response times were generally shorter than in a control condition (Jung et al., 2024).

There are also several reports on the effects of acute exercise on response inhibition. Moderate exercise has been associated with shortened stop-signal reaction times (SSRTs) together with increased P3 amplitude in the stop-signal task (SST; Chu et al., 2015), whereas other investigations have found SSRT reductions without significant ERP changes (Yu et al., 2022). The longevity of exercise-related effects also remains unclear (Joyce et al., 2009). With high-intensity exercise, some studies reported improved performance together with increased N2 and P3 amplitudes in inhibitory paradigms (Bailey et al., 2021), whereas others observed changes in earlier components without corresponding behavioral or P3 effects (Chacko et al., 2020). Overall, these findings suggest that acute exercise could influence response inhibition, but behavioral and electrophysiological indicators do not always respond consistently, and post-exercise effects may differ depending on study characteristics and measurement timing. Among potential moderators, exercise intensity is a practically important factor that can be manipulated in real-world settings.

This study aims to elucidate the transient effects of acute exercise on response inhibition based on behavioral performance indices and ERPs obtained from the SST. While there are several cognitive tasks that evaluate inhibitory control, the SST allows for quantitative evaluation of inhibition through a clear theoretical framework (Logan and Cowan, 1984) and is supported by extensive neuroscientific findings (e.g., Ramautar et al., 2004; Huster et al., 2020). Combining it with the high temporal resolution of EEG allows for a fine-grained evaluation of how exercise could influence the cognitive processes underlying executive functions and response inhibition. Additionally, three exercise intensity groups - low, moderate, and high - were compared across four phases within a single session: before exercise (control), during exercise, immediately after exercise (recovery_1), and after heart rate had recovered to near pre-exercise levels (recovery_2). Many investigations record neural activity only before and after exercise (e.g., Chu et al., 2015; Yu et al., 2022), which simplifies EEG data collection but risks masking transient electrophysiological changes during exercise and early recovery. More broadly, relatively few studies have systematically examined both exercise intensity and the temporal trajectory of recovery-phase changes in inhibitory control using EEG/ERPs within the same experimental design, particularly including measurements during exercise. Therefore, our design enables a phase-resolved evaluation of intensity-related differences in behavioral and electrophysiological indices of response inhibition.

The working hypotheses of this study were derived from prior reports suggesting that acute aerobic exercise can transiently modulate behavioral indices of response inhibition and ERP components linked to inhibitory processing, with effects that may vary as a function of intensity and measurement timing (Joyce et al., 2009; Chu et al., 2015; Bailey et al., 2021; Yu et al., 2022). We hypothesized that: (i) moderate-to-high intensity exercise, relative to the pre-exercise control phase, would improve behavioral indices of response inhibition, reflected by shorter go reaction time and shorter SSRT, with effects expected to be most evident during exercise and/or immediately after exercise and to attenuate during later recovery; (ii) stop-related P3 amplitudes would be larger during exercise and/or immediate recovery than during control, particularly at moderate-to-high intensity (Chu et al., 2015); and (iii) N2 amplitudes would show intensity- and phase-dependent modulation during exercise and early recovery relative to control, particularly at higher intensities (Bailey et al., 2021). Finally, because evidence regarding earlier sensory-attentional components is less consistent across protocols, the P2 was assessed on an exploratory basis.

## 2 Materials and methods

### 2.1 Participants

Thirty-three healthy young adults took part in the study after giving their informed written consent. All participants reported no history of a psychiatric or neurological disorder, and they all had normal or corrected-to-normal vision. However, 12 participants were excluded from all analyses due to either poor performance on the SST (e.g., RTs on correct go trials faster than on unsuccessful stop trials, a stop accuracy outside the acceptable range of 25–75%), substantial movement-related artifacts contaminating the EEG during exercise, or technical issues (Supplementary Table S1; note that some participants met multiple exclusion criteria). This resulted in a final sample size of 21 (6 females and 15 males; age: range = 18–35 years, *M* = 19.7, SD = 3.6). Two of the participants in the experiment were left-handed. The present study was approved by the Ethics Review Committee on research with human subjects, College of Industrial Technology, Nihon University (approval number S2020-009).

### 2.2 Stop-signal task

**Figure 1:**
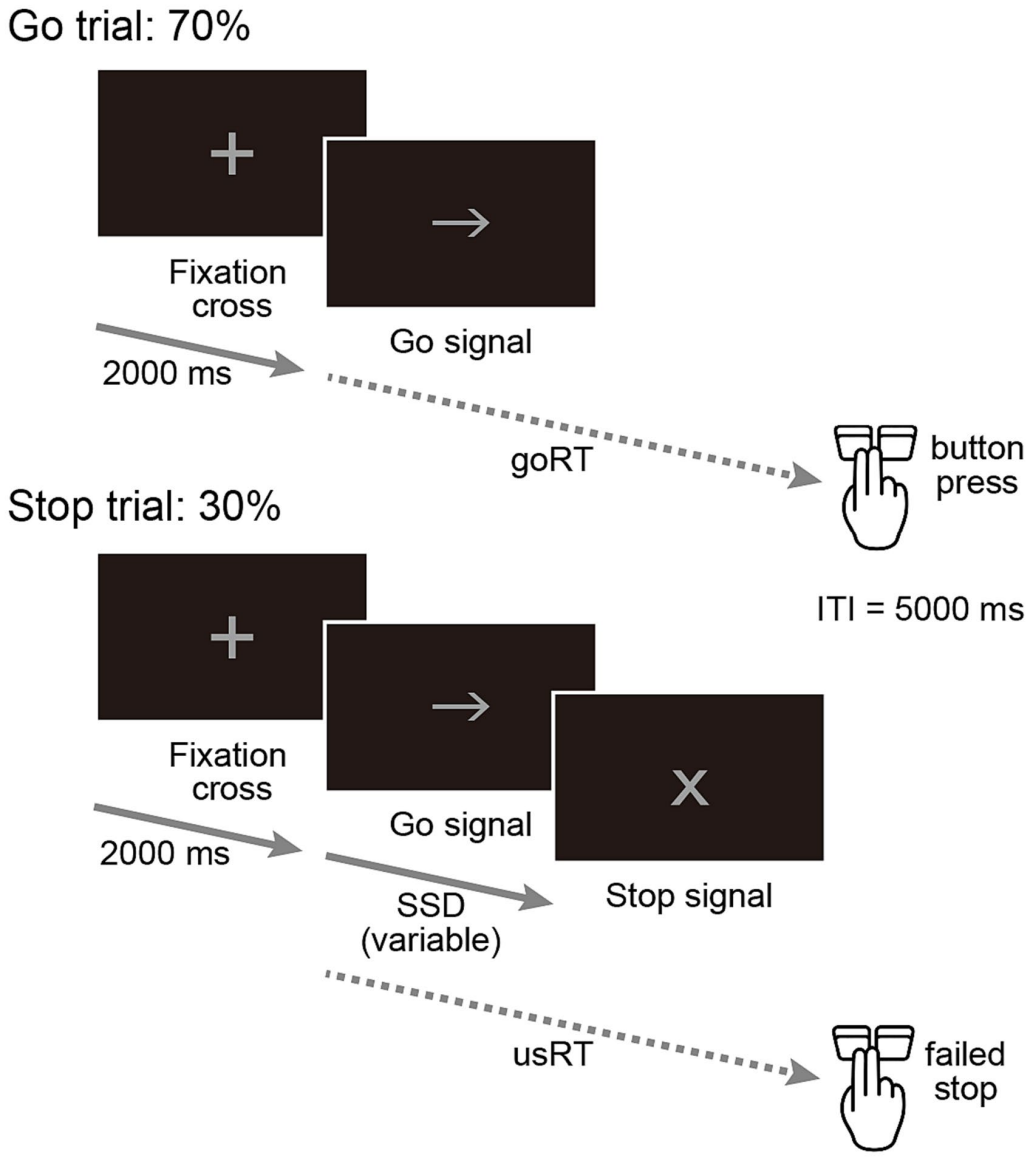
Stop-signal task in this study. Go trials (70%) and stop trials (30%) are illustrated. Each trial started with a fixation cross (2,000 ms), followed by a go signal (left- or right-pointing arrow; example shown). Participants responded with a button press as quickly as possible, and goRT was measured from go-signal onset. On stop trials, the go signal was replaced by a stop signal (“X”) after a variable stop-signal delay (SSD) relative to go-signal onset, and participants attempted to withhold their response. usRT denotes reaction time on unsuccessful-stop (failed-stop) trials. goRT was calculated from correct go trials (goCor) only; goOM and goERR were treated as accuracy measures. SSRT was estimated using the integration method (see Methods). ITI = inter-trial interval (5,000 ms); SSD = stop-signal delay.

A schematic overview of the task can be seen in Figure 1. Stimuli were presented on an Acer EB321HQ (31.5-inch screen, refresh rate 60 Hz; Acer Inc., New Taipei City, Taiwan) placed 1.5 m in front of the participant, who was seated on a cycle ergometer. The stimulus images presented had a black background, and the size of the sign, displayed in gray, was standardized to a horizontal visual angle of 3.82°. First, a cross was centrally presented as a fixation point for 2,000 ms, and then either a left or right arrow was presented as a go signal. An X-mark was presented as a stop signal 30% of the time after the go signal was presented. The stop-signal delay (SSD), the time between the onset of the go and the consecutive stop signal, started at 200 ms for each block. The SSD could vary between 100 and 600 ms, and was adjusted based on a tracking algorithm that increased or decreased the SSD by 50 ms after each successful or unsuccessful stop, respectively. In go trials, the maximum duration of the presentation of the go stimulus, which disappeared when the participant pressed the button, was 2,000 ms in addition to the corresponding SSD. Participants were instructed to respond by pressing a button on the response box with their dominant hand immediately after the go signal was presented. The inter-trial interval was fixed at 5,000 ms. The response box was placed on a platform attached under the handlebars of the cycle ergometer. Right-handed participants pressed the left-side button with the index finger of their right hand when presented with the left arrow and pressed the right-side button with the middle finger of their right hand when presented with the right arrow. For left-handed participants, the fingers that pressed the buttons were reversed. In addition, participants were instructed to cancel their responses when a stop signal was presented, but were also cautioned against waiting for the stop signal to be presented and told they should not hesitate to respond. The SST was conducted using E-Prime 3.0 software (Psychology Software Tools, Pittsburgh, PA, USA), and the Serial Response Box (Psychology Software Tools, Pittsburgh, PA, USA) recorded participant responses.

### 2.3 Acute exercise

A Monark 939E Ergomedic bicycle ergometer (Monark Exercise AB, Vansbro, Sweden) was used for acute exercise. Three exercise intensities were set according to the American College of Sports Medicine (2020) classification: low-intensity [30% heart rate reserve (HRR)], moderate-intensity (50% HRR), and high-intensity (75% HRR), and participants were divided into three groups (N_low = 6, N_moderate = 8, N_high = 7). Participants indicated their preferred exercise-intensity level at the time of registration, and group allocation was therefore based on participants’ self-selection. Heart rate (HR) was obtained by the Polar H10 sensor chest strap device (Polar Electro Oy, Kempele, Finland) and entered into the cycle ergometer. The load was manually adjusted as needed by the experimenter, who monitored the participants’ HRs and checked for signs of excessive sweating and movement during cycling. When necessary, the target workload was adjusted within the pre-defined intensity range to maintain EEG recording quality and participant safety.

### 2.4 Procedure

Participants were informed of the experiment’s setup, understood it well, and gave their consent before participating in the experiment. Participants were then prepared for EEG and HR data collection prior to practicing 1 block of the SST. The experiment was conducted with participants seated on the cycle ergometer. First, the participants were placed in a seated resting position for 1 min and their resting HR was recorded, then SST was performed in the control condition. After this, participants cycled for 3 min to reach and maintain their target exercise intensity as a warm-up, after which the SST was performed in the exercise condition. After completing the SST during exercise, participants continued exercising for 1 min and then underwent SST under the recovery_1 condition. The starting time for the SST in the recovery_2 condition was defined as the point at which HR decreased to within 120% of resting HR after the end of the recovery_1 condition. Once the last condition was completed, all electrodes were removed. Each participant thus completed the SST four times in different conditions, and each SST was conducted in two blocks of 50 trials, with a 1-min interblock interval. Note that to keep task blocks short and thus minimize the effect of sweating on EEG recordings during exercise, the number of SST trials in this study was lower than recommended by Verbruggen et al. (2019).

### 2.5 EEG recording

EEG data were recorded using the eego™ sports system (ANT Neuro B. V., Hengelo, The Netherlands) with 32 Ag/AgCl electrodes positioned according to the international 10–20 system. CPz was used as the recording reference and AFz served as the ground electrode. The sampling rate was set at 1,000 Hz, and electrode impedances were kept below 20 kΩ throughout the recording (Park and Donaldson, 2019). The DC-coupled amplifier allowed full-bandwidth acquisition from 0 Hz (DC) to approximately 262 Hz, limited by the system’s anti-aliasing filter.

### 2.6 Behavioral analyses

The following variables were calculated as behavioral performance indicators: RTs in correct go trials (goRT), RTs in unsuccessful stop trials (usRT), the stop-signal reaction time (SSRT), the stop-signal delay (SSD), the percentage of go omissions (goOM), erroneous go responses (goERR), and the stopping accuracy (stopACC). RTs were calculated as the time elapsed from the onset of the go signal until a response was recorded. Trials with RTs longer than 1,000 ms were excluded from the analysis, and trials with RTs outside 3.5 SD of each participant’s mean RT were also excluded as outliers. SSRT was calculated using the integration method based on the full RT distributions, with go omissions replaced (Verbruggen et al., 2019). goOM and goERR represent the percentage of go trials with no response or an incorrect response, respectively. StopACC indicates the percentage of stop trials in which the response was successfully withheld.

### 2.7 EEG processing

EEG data were preprocessed offline using EEGLAB (v. 2025.0.0; Delorme and Makeig, 2004), a MATLAB-based open-source toolbox. The data were re-referenced to the common average of all electrodes, excluding the mastoid electrodes (M1 and M2). A high-pass filter at 1.5 Hz and a low-pass filter at 25 Hz were applied to reduce exercise-related artifacts, which is a narrower frequency band than typically used. This relatively narrow filter setting was chosen as a pragmatic compromise for exercise EEG to attenuate slow drifts (e.g., sweating- and movement-related) and high-frequency EMG contamination during cycling while preserving the ERP components of interest, including the P3. The same high-pass and low-pass settings were applied to all conditions and groups. The data were then resampled to 500 Hz. Continuous data were segmented into epochs time-locked to the onset of the fixation cue (S1), spanning from −1,000 ms to 4,000 ms relative to fixation onset. Independent component analysis (ICA) was then performed using the extended Infomax algorithm (Lee et al., 1999) to identify and remove components associated with eye movements, muscle activity, and other non-neural artifacts. The number of removed ICA components are summarized in Supplementary Table S3. For transparency, epoch counts after preprocessing (numbers of rejected and retained epochs and retention rates) by phase, trial type (goCor, SStop, UStop), and intensity group are reported in Supplementary Table S2. For each exercise condition, preprocessed data were then re-segmented with a time-window of −200 to 1,000 ms time-locked to go signal onset in go trials and stop signal onset in stop trials. ERP amplitudes related to correct go responses (goCor) were derived from epochs time-locked to go signal onset, while ERPs for stop trials were computed separately for successful (SStop) and unsuccessful (UStop) stop trials. Baseline correction was performed using the −200 ms to 0 ms pre-stimulus interval. No additional amplitude normalization across conditions was applied. Subsequently, automatic artifact rejection was applied using the pop_autorej function in EEGLAB to exclude epochs containing artifacts. ERP amplitudes (P2, N2, P3) were quantified using mean amplitudes of small windows centered at individually estimated peaks, which reduces sensitivity to single-sample fluctuations and minor latency jitter. P2 was measured at Fz, and N2 and P3 at Cz. The following time windows were used to localize the different ERP peaks for each individual and condition: P2 (100–250 ms), N2 (150–300 ms), and P3 (250–500 ms), relative to stimulus onset. The polarity for each component was set according to standard conventions (negative for N2, positive for P2/P3), and the mean peak was calculated from a window centered around the estimated ERP peak (P2 = 40 ms, N2 = 60 ms, P3 = 100 ms). Peak latencies for P2, N2, and P3 were extracted for descriptive purposes. For each participant, condition, and trial type, the peak latency was defined as the time point of the component peak within the same pre-defined time window used for the mean peak amplitude. Latency values were summarized as mean plus or minus SD and are reported in Supplementary Table S4 without inferential statistical testing.

### 2.8 Statistical analysis

To verify whether the target exercise intensities (%HRR) were successfully implemented across the three groups (low, moderate, high), a one-way ANOVA was conducted. The dependent variable was the %HRR calculated from average HR during the exercise condition, and the independent variable was the exercise intensity group. Levene’s test was used to assess the assumption of homogeneity of variances. When a significant main effect was found, Tukey’s HSD *post hoc* tests were performed. Effect sizes were reported using η^2^ for the ANOVA and Cohen’s d for post hoc comparisons.

To examine the effects of exercise intensity on behavioral performance in the SST, a series of 3 (group: low, moderate, high) × 4 (condition: control, exercise, recovery_1, recovery_2) mixed-design ANOVAs were conducted separately for each of the seven performance indices: goRT, usRT, SSRT, SSD, goOM, goERR, and stopACC. The within-subjects factor was condition, and the between-subjects factor was group. Mauchly’s test of sphericity was applied to assess the assumption of sphericity for within-subjects effects, and Greenhouse–Geisser corrections were applied when the assumption was violated. When significant main effects or interactions were identified, Bonferroni-corrected *post hoc* comparisons were conducted to examine pairwise differences. Effect sizes were reported using partial eta squared (η^2^).

To examine the effects of exercise intensity on ERP amplitudes, 3 (group: low, moderate, high) × 4 (condition: control, exercise, recovery_1, recovery_2) mixed-design ANOVAs were conducted separately for the mean peak amplitude of each ERP component (P2, N2, and P3). The within-subjects factor was condition, and the between-subjects factor was group. To limit multiple comparisons in the ERP analyses, statistical inference was restricted *a priori* to predefined ERP components, electrodes, and time windows (P2 at Fz; N2 and P3 at Cz), and post hoc pairwise comparisons were Bonferroni-corrected. Mauchly’s test of sphericity was performed to evaluate the assumption of sphericity for within-subjects effects, and Greenhouse–Geisser corrections were applied when the assumption was violated. When significant main effects or interactions were observed, Bonferroni-corrected post hoc comparisons were conducted to assess pairwise differences. Effect sizes were reported using partial eta squared (η^2^). In addition, to provide a robustness check while keeping the number of additional tests limited, we conducted non-parametric sign-flip permutation tests with 5,000 permutations for two a priori contrasts central to our conclusions: goCor N2 (Exercise minus Control) and UStop P3 (Recovery_1 minus Control). For each contrast, participant-level difference scores were computed and randomly sign-flipped to generate an empirical null distribution and a two-sided *p* value. Most statistical analyses were conducted using JASP (v. 0.19.3.0); permutation tests were conducted using R (v. 4.5.1).

## 3 Results

### 3.1 HRR results

Table 1 shows the HR for each condition. The mean %HRR during exercise for each group was low: 32.2% (SD = 3.8), moderate: 47.1% (SD = 2.4), high: 65.4% (SD = 4.0). A one-way ANOVA revealed a significant effect of group on %HRR, *F*(2, 18) = 153.92, *p* < 0.001, η^2^ = 0.945. *Post hoc* Tukey tests indicated that all groups differed significantly from each other (all *p* < 0.001), confirming that the target exercise intensities (low, moderate, high) were successfully implemented and distinguishable. The effect sizes were very large across all comparisons (Cohen’s d > 4.3). Although the high-intensity group did not reach the target level of 75% HRR, the achieved intensity still fell within the vigorous-intensity range defined by the American College of Sports Medicine (2020) guidelines (60–89% HRR), and a significant difference was observed among the three groups.

**Table 1:** Heart rate (bpm) across phases and achieved exercise intensity (%HRR) during the exercise phase.

Phase	Low intensity (SD)	Moderate intensity (SD)	High intensity (SD)
HR			
Rest	82.8 (3.5)	76.7 (2.6)	80.2 (3.4)
Control	83.4 (3.8)	77.2 (3.1)	80.8 (3.6)
Exercise	120.8 (3.6)	134.4 (3.4)	159.3 (3.8)
Recovery_1	89.7 (6.2)	90.6 (11.8)	108.6 (10.9)
Recovery_2	83.3 (3.3)	85.0 (3.1)	92.5 (3.6)
%HRR			
Exercise	32.2 (3.8)	47.1 (2.4)	65.4 (4.0)

Values are mean (SD). %HRR was calculated from the mean heart rate during the exercise phase.

### 3.2 SST behavioral results

**Figure 2:**
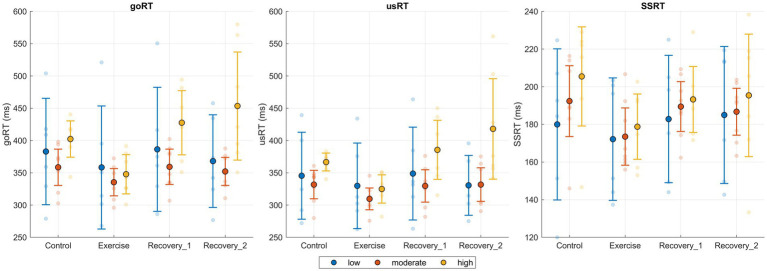
goRT, usRT, and SSRT across experimental phases and exercise intensity groups. Dots indicate individual participants, larger circles show group means, and error bars denote 95% confidence intervals. Phases include Control, Exercise, Recovery_1, and Recovery_2. Because intensity was a between-subject factor, between-group differences in absolute values should be interpreted cautiously; statistical inferences are reported in the text.

Figure 2 shows goRT, usRT, and SSRT across conditions with individual participant data points and the overall mean with 95% confidence intervals, providing a descriptive overview of the magnitude and variability of behavioral performance. Table 2 presents descriptive statistics for each group and condition across the seven performance indices of the SST: goRT, usRT, SSRT, SSD, goOM, goERR, and stopACC. To examine the effects of exercise condition and group, 3 (group: low, moderate, high) × 4 (condition: control, exercise, recovery_1, recovery_2) mixed-design ANOVAs were conducted for each variable. For all analyses, Mauchly’s test indicated that the assumption of sphericity was violated for relevant within-subjects effects (*p* < 0.05), and therefore, Greenhouse–Geisser corrections were applied to the degrees of freedom. *Post hoc* comparisons were conducted using Bonferroni correction.

**Table 2:** Stop-signal task behavioral performance across phases and exercise-intensity groups.

Phase	Low intensity (SD)	Moderate intensity (SD)	High intensity (SD)
goRT (ms)			
Control	383.00 (70.54)	358.56 (53.87)	402.34 (69.80)
Exercise	358.38 (51.64)	335.42 (56.15)	347.82 (48.80)
Recovery_1	386.36 (63.01)	359.29 (57.72)	427.61 (75.57)
Recovery_2	368.15 (55.83)	352.10 (51.18)	453.51 (83.63)
usRT (ms)			
Control	345.47 (41.57)	331.77 (40.57)	366.65 (46.60)
Exercise	329.81 (36.97)	309.64 (37.85)	324.91 (36.74)
Recovery_1	348.80 (46.97)	329.66 (39.57)	385.54 (42.87)
Recovery_2	330.59 (45.60)	331.74 (40.68)	418.10 (53.48)
SSRT (ms)			
Control	180.00 (38.28)	192.38 (22.55)	205.48 (28.50)
Exercise	172.17 (31.01)	173.50 (18.23)	178.76 (18.77)
Recovery_1	182.83 (32.24)	189.46 (15.83)	193.29 (18.95)
Recovery_2	185.00 (34.65)	186.75 (14.87)	195.43 (35.18)
SSD (ms)			
Control	186.67 (52.21)	161.25 (45.74)	185.24 (52.98)
Exercise	181.67 (48.90)	154.38 (41.10)	166.67 (42.28)
Recovery_1	194.17 (50.85)	165.42 (44.93)	220.71 (54.22)
Recovery_2	182.50 (43.86)	162.50 (45.00)	231.43 (53.03)
goOM (%)			
Control	0.00 (0.00)	0.18 (0.51)	0.21 (0.55)
Exercise	0.00 (0.00)	0.00 (0.00)	0.00 (0.00)
Recovery_1	0.00 (0.00)	0.00 (0.00)	0.00 (0.00)
Recovery_2	0.00 (0.00)	0.00 (0.00)	1.85 (4.28)
goERR (%)			
Control	0.49 (0.75)	0.36 (1.02)	0.00 (0.00)
Exercise	2.63 (3.77)	1.44 (1.34)	0.41 (0.71)
Recovery_1	0.00 (0.00)	0.37 (0.69)	0.20 (0.54)
Recovery_2	0.72 (1.21)	0.36 (1.01)	1.85 (4.28)
stopACC (%)			
Control	50.00 (8.94)	46.25 (4.52)	49.52 (2.30)
Exercise	45.56 (9.81)	46.25 (2.78)	46.19 (4.88)
Recovery_1	48.33 (9.60)	46.25 (4.86)	49.52 (4.88)
Recovery_2	46.67 (5.96)	46.25 (2.14)	53.81 (7.05)

Values are mean (SD). goRT, reaction time in correct go trials; usRT, reaction time in unsuccessful stop trials; SSRT, stop-signal reaction time; SSD, stop-signal delay (all in ms). goOM, go omissions; goERR, erroneous go responses; stopACC, stopping accuracy (all in %).

In goRT, there was a significant main effect of condition, *F*(3, 54) = 10.53, *p* < 0.001, η^2^ = 0.080, and a significant condition × group interaction, *F*(6, 54) = 4.15, *p* = 0.002, η^2^ = 0.063. The main effect of group was not significant, *F*(2, 18) = 2.22, *p* = 0.138, η^2^ = 0.142. Post hoc comparisons across all participants showed that RTs were significantly faster in the exercise condition compared to control (*p* < 0.001), recovery_1 (*p* < 0.001), and recovery_2 (*p* = 0.008). In addition, simple effects analysis within each group revealed that in the high-intensity group, goRT in the exercise condition was significantly shorter than in control (*p* = 0.002), recovery_1 (*p* < 0.001), and recovery_2 (*p* = 0.003).

In usRT, a significant main effect of condition, *F*(3, 54) = 7.35, *p* < 0.001, η^2^ = 0.080, and a significant condition × group interaction, *F*(6, 54) = 3.46, *p* = 0.006, η^2^ = 0.075, were observed. The main effect of group was not significant, *F*(2, 18) = 2.90, *p* = 0.081, η^2^ = 0.158. *Post hoc* comparisons across all participants indicated that usRTs were significantly shorter in the exercise condition than in control (*p* < 0.001), recovery_1 (*p* < 0.001), and recovery_2 (*p* = 0.013). Additionally, within the high-intensity group, usRT was significantly shorter in the exercise condition compared to control (*p* = 0.001), recovery_1 (*p* = 0.002), and recovery_2 (*p* = 0.013). A significant difference was also found in the moderate-intensity group, where usRT was significantly shorter in the exercise condition compared to recovery_2 (*p* = 0.032).

In SSRT, there was a significant main effect of condition, *F*(3, 54) = 3.47, *p* = 0.022, η^2^ = 0.068, with *post hoc* comparisons revealing that SSRT was significantly shorter in the exercise condition compared to control (*p* = 0.011). Neither the main effect of group (*F*(2, 18) = 0.71, *p* = 0.504, η^2^ = 0.041) nor the condition × group interaction (*F*(6, 54) = 0.34, *p* = 0.916, η^2^ = 0.013) was statistically significant.

In SSD, a significant main effect of condition was found, *F*(3, 54) = 3.43, *p* = 0.023, η^2^ = 0.054. Post hoc comparisons revealed that SSD was significantly longer in recovery_1 compared to exercise (*p* = 0.007). Neither the main effect of group (*F*(2, 18) = 2.80, *p* = 0.087, η^2^ = 0.142) nor the condition × group interaction (*F*(6, 54) = 1.91, *p* = 0.095, η^2^ = 0.061) was statistically significant.

In goOM, no significant effects were observed for condition (*F*(3, 54) = 1.11, *p* = 0.352, η^2^ = 0.040), group (*F*(2, 18) = 1.49, *p* = 0.251, η^2^ = 0.033), or the condition × group interaction (*F*(6, 54) = 1.21, *p* = 0.315, η^2^ = 0.086).

In goERR, a trend toward a main effect of condition was found, *F*(3, 54) = 2.77, *p* = 0.051, η^2^ = 0.085. Neither the main effect of group (*F*(2, 18) = 0.24, *p* = 0.793, η^2^ = 0.007) nor the condition × group interaction (*F*(6, 54) = 1.41, *p* = 0.228, η^2^ = 0.087) was statistically significant.

Lastly, in stopACC, no significant effects were observed for condition (*F*(3, 54) = 2.56, *p* = 0.092, η^2^ = 0.035), group (*F*(2, 18) = 1.00, *p* = 0.391, η^2^ = 0.062), or the condition × group interaction (*F*(6, 54) = 1.85, *p* = 0.107, η^2^ = 0.058).

Overall, the observed behavioral effects were medium in magnitude (partial η^2^ values approximately 0.05–0.08 for the significant condition effects), supporting a transient facilitation during exercise.

### 3.3 ERP results

**Figure 3:**
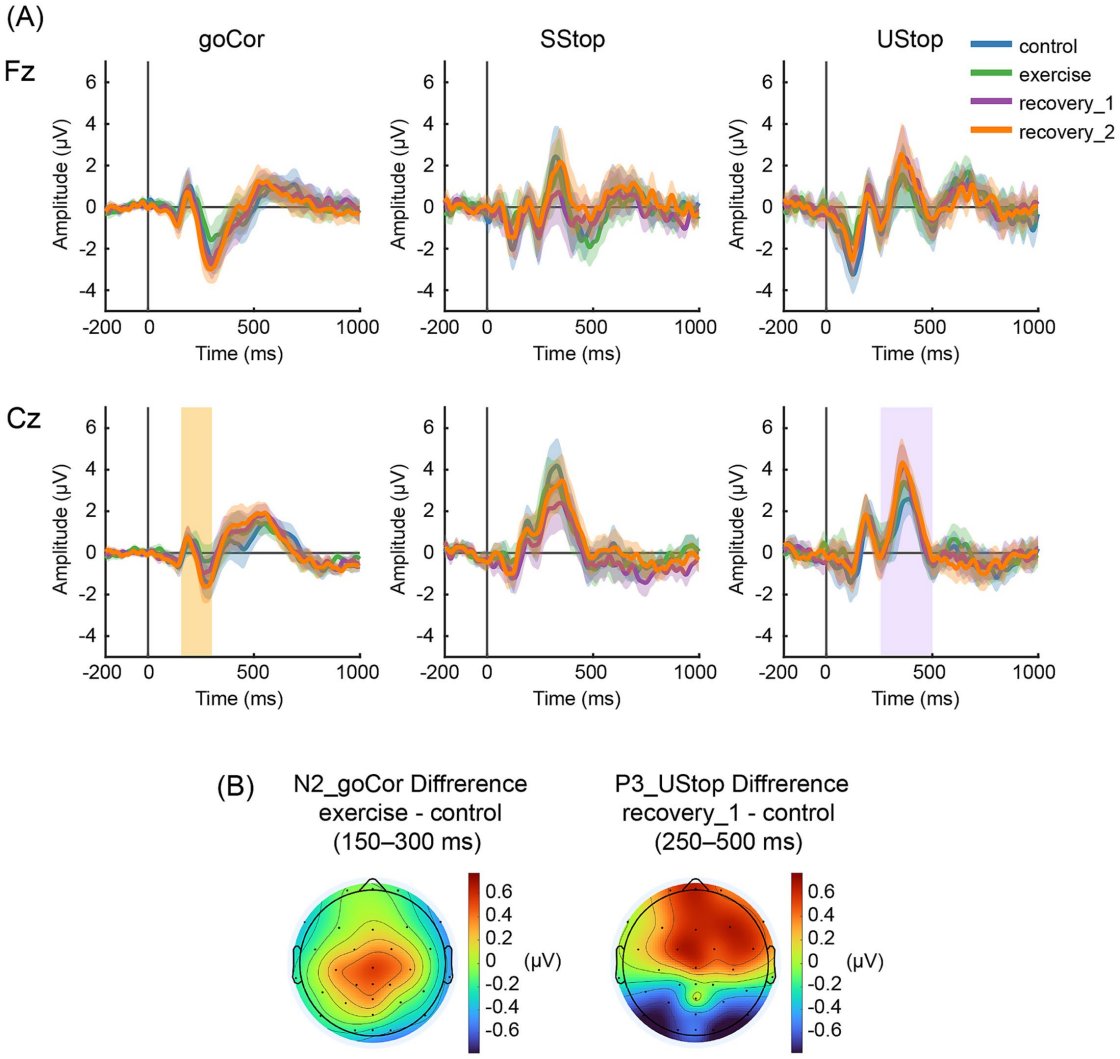
Group-averaged ERP waveforms and difference topographies. **(A)** Group average ERP waveforms from Fz and Cz electrodes. The shaded areas represent SEM. “goCor” indicates the group average waveform during correct responses in go trials. “SStop” indicates the group average waveform during successful stop trials, while “UStop” indicates the group average waveform during unsuccessful stop trials. The orange band indicates the predefined latency window used for N2 amplitudes quantification (corresponding to the component showing a significant condition effect). The purple band indicates the predefined latency window used for P3 amplitudes quantification (corresponding to the component showing a significant condition effect). **(B)** Scalp topographies of grand-average difference waves illustrating the spatial distribution of the significant effects: goCor N2 difference (exercise minus control) and UStop P3 difference (recovery_1 minus control).

Figure 3A shows the ERP waveforms for Fz and Cz for each condition, and Figure 3B shows scalp topographies for the primary contrasts to illustrate the spatial distribution of the effects. ERP amplitudes related to the goCor were derived from epochs time-locked to the onset of the go signal, and ERP amplitudes related to SStop and UStop were derived from epochs time-locked to the onset of the stop signal. Retained and rejected epoch counts (and retention rates) contributing to ERP averages for each phase and trial type are provided in Supplementary Table S2. Table 3 shows the average amplitude of each component obtained by the mean peak amplitude method. The P2 amplitude was obtained from Fz, while the N2 and P3 amplitudes were obtained from Cz. For completeness, descriptive peak latency values for P2, N2, and P3 across all trial types and conditions are provided in Supplementary Table S4.

**Table 3:** Mean peak ERP amplitudes (μV) of P2 (Fz), N2 (Cz), and P3 (Cz) by trial type, phase, and exercise-intensity group.

ERP component (electrode)	Trial type	Phase	Low intensity (SD)	Moderate intensity (SD)	High intensity (SD)
P2 (Fz)	goCor	Control	1.55 (1.29)	1.74 (1.60)	1.34 (0.38)
		Exercise	0.91 (0.47)	1.00 (0.48)	1.36 (0.49)
		Recovery_1	0.79 (1.21)	1.49 (1.35)	1.23 (0.90)
		Recovery_2	0.80 (1.06)	1.18 (1.40)	1.16 (0.64)
	SStop	Control	1.54 (2.04)	1.55 (1.35)	0.76 (0.91)
		Exercise	1.15 (3.62)	1.71 (1.56)	1.55 (1.52)
		Recovery_1	1.88 (2.04)	0.70 (1.10)	0.74 (1.40)
		Recovery_2	1.97 (1.05)	0.99 (0.90)	0.96 (2.18)
	UStop	Control	0.83 (1.65)	1.39 (1.29)	0.17 (1.79)
		Exercise	0.89 (1.91)	2.38 (2.00)	0.77 (2.36)
		Recovery_1	1.54 (2.04)	1.57 (1.27)	1.03 (1.27)
		Recovery_2	1.16 (2.58)	1.17 (1.14)	1.08 (1.87)
N2 (Cz)	goCor	Control	−1.88 (1.35)	−2.04 (1.02)	−1.66 (0.94)
		Exercise	−0.78 (0.93)	−1.25 (0.58)	−0.82 (0.75)
		Recovery_1	−1.39 (0.96)	−1.62 (1.22)	−1.64 (0.85)
		Recovery_2	−1.96 (1.25)	−1.94 (1.37)	−1.82 (1.02)
	SStop	Control	−0.66 (1.13)	−0.06 (1.74)	−0.46 (1.12)
		Exercise	0.01 (0.54)	−0.34 (1.69)	−0.03 (1.22)
		Recovery_1	−0.83 (1.49)	−0.76 (1.62)	−0.97 (1.01)
		Recovery_2	−0.12 (1.71)	−0.86 (1.40)	0.06 (2.24)
	UStop	Control	−1.01 (1.28)	−1.10 (1.29)	−1.23 (1.18)
		Exercise	−0.78 (1.51)	0.09 (1.18)	−1.42 (1.51)
		Recovery_1	−0.77 (1.34)	−0.77 (1.10)	−1.47 (1.53)
		Recovery_2	−0.61 (1.73)	−1.22 (1.51)	−0.64 (1.40)
P3 (Cz)	goCor	Control	1.17 (1.29)	1.65 (1.29)	0.94 (0.88)
		Exercise	1.24 (0.56)	2.07 (1.26)	1.15 (0.66)
		Recovery_1	1.54 (1.47)	2.60 (1.54)	1.06 (1.38)
		Recovery_2	1.70 (1.67)	2.55 (0.81)	1.36 (1.11)
	SStop	Control	4.52 (1.86)	4.46 (2.12)	3.31 (1.95)
		Exercise	3.47 (2.42)	3.72 (1.54)	2.92 (1.82)
		Recovery_1	3.24 (2.09)	2.88 (1.58)	2.83 (1.14)
		Recovery_2	3.88 (2.57)	3.10 (1.56)	3.91 (2.03)
	UStop	Control	2.42 (1.16)	3.06 (1.22)	2.69 (1.29)
		Exercise	3.58 (1.49)	4.17 (1.71)	1.96 (1.82)
		Recovery_1	4.01 (1.84)	3.55 (1.89)	3.50 (1.50)
		Recovery_2	3.92 (2.25)	3.34 (1.34)	3.91 (1.66)

Values are mean (SD). goCor, correct go trials; SStop, successful stop trials; UStop, unsuccessful stop trials. N2 amplitudes are negative by convention.

#### 3.3.1 P2

In goCor, a mixed-design ANOVA revealed no significant main effect of condition, *F*(3, 54) = 2.252, *p* = 0.093, η^2^ = 0.033, nor a significant main effect of group, *F*(2, 18) = 0.229, *p* = 0.798, η^2^ = 0.017. There was also no significant condition × group interaction, *F*(6, 54) = 0.700, *p* = 0.651, η^2^ = 0.020.

Similarly, no significant main effect of condition, *F*(3, 54) = 0.155, *p* = 0.926, η^2^ = 0.005, or group, *F*(2, 18) = 0.506, *p* = 0.611, η^2^ = 0.020, nor a condition × group interaction, *F*(6, 54) = 0.492, *p* = 0.812, η^2^ = 0.033, were observed for the P2 amplitude measured during successful stop trials.

In UStop, no significant effects were found for condition, *F*(3, 54) = 0.599, *p* = 0.619, η^2^ = 0.015, group, *F*(2, 18) = 0.761, *p* = 0.482, η^2^ = 0.039, or the condition × group interaction, *F*(6, 54) = 0.506, *p* = 0.801, η^2^ = 0.026, for P2 amplitude during unsuccessful stop trials.

#### 3.3.2 N2

In goCor, a mixed-design ANOVA revealed a significant main effect of condition, *F*(3, 54) = 10.014, *p* < 0.001, η^2^ = 0.114, but no significant main effect of group, *F*(2, 18) = 0.125, *p* = 0.883, η^2^ = 0.009, nor a condition × group interaction, *F*(6, 54) = 0.384, *p* = 0.886, η^2^ = 0.009. *Post hoc* comparisons indicated significantly smaller N2 amplitudes in the exercise condition compared to both the control (*p* < 0.001) and recovery_1 (*p* = 0.015) conditions. Additionally, the N2 amplitude was significantly smaller in exercise than in recovery_2 (*p* = 0.002). A non-parametric sign-flip permutation test for the primary contrast confirmed the goCor N2 difference between exercise and control (*p*_perm < 0.0002).

In SStop, no significant main effects were found for condition, *F*(3, 54) = 1.181, *p* = 0.326, η^2^ = 0.031, group, *F*(2, 18) = 0.037, *p* = 0.964, η^2^ = 0.002, or the condition × group interaction, *F*(6, 54) = 0.505, *p* = 0.802, η^2^ = 0.026.

Similarly, in UStop, no significant effects were observed for condition, *F*(3, 54) = 0.497, *p* = 0.686, η^2^ = 0.012, group, *F*(2, 18) = 0.361, *p* = 0.702, η^2^ = 0.019, or the condition × group interaction, *F*(6, 54) = 1.114, *p* = 0.366, η^2^ = 0.054, for N2 amplitude during unsuccessful stop trials.

#### 3.3.3 P3

In goCor, a mixed-design ANOVA revealed a significant main effect of condition, *F*(3, 54) = 2.886, *p* = 0.044, η^2^ = 0.031, but no significant main effect of group, *F*(2, 18) = 1.861, *p* = 0.184, η^2^ = 0.131, nor a condition × group interaction, *F*(6, 54) = 0.499, *p* = 0.806, η^2^ = 0.011. *Post hoc* tests revealed no significant pairwise differences between conditions after Bonferroni correction.

In SStop, a mixed-design ANOVA indicated no significant main effect of condition (*F*(3, 54) = 2.390, *p* = 0.079, η^2^ = 0.042), no significant main effect of group (*F*(2, 18) = 0.169, *p* = 0.846, η^2^ = 0.011), and no significant condition × group interaction (*F*(6, 54) = 0.782, *p* = 0.588, η^2^ = 0.027) for P3 amplitude in successful stop trials.

In UStop, a mixed-design ANOVA revealed a significant main effect of condition (*F*(3, 54) = 3.931, *p* = 0.013, η^2^ = 0.053) and condition × group interaction (*F*(6, 54) = 2.695, *p* = 0.023, η^2^ = 0.072). The main effect of group was not significant (*F*(2, 18) = 0.260, *p* = 0.774, η^2^ = 0.018). Post hoc comparisons indicated significantly smaller P3 amplitudes in the control compared to the recovery_1 condition (*p* = 0.009). No other significant differences emerged after Bonferroni correction. A non-parametric sign-flip permutation test for the primary contrast also supported the UStop P3 difference between recovery_1 and control (*p*_perm = 0.0014).

## 4 Discussion

The working hypothesis of this study was that moderate to high-intensity acute exercise would transiently improve response inhibition, reflected in both behavioral and neural measures, and that these effects would vary depending on exercise intensity and the timing of assessment (control, exercise, recovery_1, recovery_2). Overall, the results supported some of the hypotheses, but the changes in ERP components were selective and differed partly from the predictions.

### 4.1 Summary of main findings

Behaviorally, we found significant main effects of condition for both goRT and usRT. Collapsed across groups, *post hoc* comparisons showed that responses were significantly shorter during the exercise condition than during the control condition, and then significantly lengthened in recovery_1 and recovery_2 relative to exercise, typically returning to or even exceeding control levels. Effect sizes and post hoc patterns further suggest that this main effect was mostly driven by the more strenuous conditions, with the high-intensity group showing the most pronounced in-exercise reductions. However, interpretations of intensity-dependent patterns should be made cautiously because intensity was manipulated between participants and group allocation was based on self-selection, and because the final sample was small and sex-imbalanced (*N* = 21; 6 females). These factors limit power—especially for interactions—and may constrain generalizability; future studies with larger, more sex-balanced samples and randomized allocation or within-subject crossover designs will be important to strengthen causal inference. Thus, the behavioral facilitation of acute exercise appeared to be largely confined to the period during exercise.

A significant main effect of condition was also observed in SSRTs, with the exercise condition showing a significantly shorter SSRT than the control condition. In other words, acute exercise had a positive effect on both the go process of response execution and the stop process of response inhibition. No statistically significant main effects or interactions were observed for goOM, goERR, or stopACC, suggesting that the exercise-related improvements in reaction speed and SSRT were not accompanied by clear accuracy costs in the present data.

Electrophysiologically, acute exercise showed selective effects across components and trial types. For the P2, no significant main effects or interactions were observed in either go or stop trials. For the N2, a significant main effect of condition was observed in goCor, with smaller amplitudes during exercise than during control (and smaller than during both recovery conditions). In stop trials, neither SStop-N2 nor UStop-N2 showed significant exercise-related effects. For P3, effects were limited: goCor-P3 showed a small condition main effect without pairwise differences surviving *post hoc* correction, and SStop-P3 showed no significant effects. In UStop, P3 amplitude was larger in recovery_1 than in control at Cz, and the primary contrast was supported by a sign-flip permutation test. Because each SST phase was kept relatively short to minimize sweating and movement during cycling and to maintain EEG recording quality, resulting in trial counts below some best-practice recommendations (Verbruggen et al., 2019), the number of trials contributing to SSRT and ERP estimates—especially for stop trials—was limited.

With respect to our *a priori* hypotheses, Hypothesis (i) was supported in that behavioral facilitation was evident during exercise, but we did not observe clear carryover into recovery_1 or recovery_2. Hypothesis (ii) received only limited support: we observed a modest stop-related P3 increase in early recovery for unsuccessful stops, but not during exercise and not for successful stops. Hypothesis (iii) was partially supported in that N2 showed phase-dependent modulation (goCor-N2 reduction during exercise), but this modulation was not clearly intensity-dependent and did not extend to stop-related N2 measures. Finally, consistent with its exploratory status, P2 did not show reliable exercise-related modulation in this protocol.

### 4.2 Mechanistic interpretations

Cognitive tasks are known to involve a speed–accuracy tradeoff (SAT) (Ratcliff and McKoon, 2008). In the present study, the absence of statistically significant changes in goOM, goERR, and stopACC suggests that faster goRT/usRT and shorter SSRT during exercise were not accompanied by clear accuracy costs. Future studies with larger samples and more trials could apply diffusion-model analyses (e.g., drift–diffusion modeling) to dissociate changes in decision threshold, drift rate, and non-decision time underlying the exercise-related RT/SSRT effects.

One possible account is that acute exercise transiently increases arousal and neuromodulatory drive, which could enhance signal-to-noise characteristics and facilitate executive processing. This phenomenon aligns with the catecholamine hypothesis (McMorris, 2016), which posits that acute exercise activates the locus coeruleus, increasing the release of norepinephrine and dopamine. These catecholamines enhance the signal-to-noise ratio in the prefrontal cortex, thereby facilitating executive functions. Studies utilizing on fNIRS for neuroimaging (e.g., Ji et al., 2019) have shown increased prefrontal cortex oxygenation during cognitive tasks following acute exercise, suggesting that such hemodynamic changes may contribute to faster responses and improved executive function. At the same time, because we did not directly measure catecholamines or cerebral hemodynamics, mechanistic interpretations in these terms should be regarded as provisional and should be tested in future work with appropriate physiological measures.

Among the ERP components we examined, the P2 component reflects earlier stages of stimulus processing—perceptual and attentional processing—while the N2 and P3 components reflect the subsequent cognitive control stage (Crowley and Colrain, 2004; Folstein and Van Petten, 2008; Finnigan et al., 2011; Huster et al., 2020). Regardless of task condition or exercise intensity, we found no effect of exercise on the P2 amplitude. Previous work (Chen et al., 2021) found that a reduction in P2 amplitude is indicative of a decreased initial orienting response or impaired allocation of attentional resources. In the SST, increased P2 amplitudes have been reported in successful inhibition trials (Senderecka et al., 2012), suggesting a link between attentional allocation and inhibition performance. Prior studies investigating the effects of acute exercise on SST performance have not explicitly addressed the P2 component. The present findings suggest that acute exercise does not substantially affect the initial perceptual and attentional processing stages of stimulus processing; rather, it selectively influences the subsequent cognitive control stages. This represents important evidence supporting the notion that the impact of acute exercise is not uniform across all cognitive processes but rather selective to specific hierarchical stages within the processing pipeline.

The N2 component is thought to reflect activation of the frontal cortex, particularly the anterior cingulate cortex (ACC), associated with cognitive conflict and response monitoring during response selection, as well as with processes underlying efficient response preparation and focused attention (Folstein and Van Petten, 2008). Within the SST, the N2 component is typically observed in response to the stop signal, whereas the N2 elicited by go stimuli tends to be less prominent but may still be present. Generally, the N2 amplitude for go trials is smaller compared to stop trials due to the absence of inhibitory demands [but note that the N2 is unlikely to serve as a direct marker of response inhibition itself (Huster et al., 2020)]. However, certain participant factors may lead to altered N2 amplitudes also for go stimuli; for instance, individuals with ADHD tendencies have been reported to exhibit larger N2 amplitudes in response to go stimuli compared to healthy controls (Johnstone et al., 2007). In the present study, a significant reduction in N2 amplitude was observed during exercise in the ERP elicited by go stimuli, mirroring our findings of faster goRTs during exercise. This pattern indicates that acute exercise modulated N2-indexed processes engaged during go processing. Although conservative preprocessing was applied, residual movement/EMG contributions during cycling cannot be fully excluded; therefore, exercise-phase ERP differences (including the goCor-N2 reduction) should be interpreted cautiously. However, reduced N2 amplitude does not uniquely imply “improved efficiency”; it may also reflect attenuated engagement of conflict monitoring processes, decreased neural recruitment, or other shifts in the allocation of control-related processing. The stop-N2 is commonly interpreted as reflecting conflict monitoring and detection, reflecting the conflicting performance demands that arise when an ongoing go process is faced with a subsequent stop signal (Ramautar et al., 2004; Folstein and Van Petten, 2008). In the present study, neither the SStop-N2 nor the UStop-N2 showed any significant effect of exercise nor exercise intensity, suggesting that, within the sensitivity of our measures, acute exercise did not measurably alter stop-related conflict monitoring indexed by the stop-N2.

Overall, the pattern of changes in goCor-N2 amplitudes is consistent with the changes in reaction times, suggesting that the reaction time improvement could be facilitated by altered engagement of go-related control processes in go-related response preparation/selection and reduced conflict during go processing. The lack of measurable changes in stop-related N2 amplitudes, however, suggests that the observed SSRT reduction during exercise could arise through a different underlying mechanism. Taken together, these findings indicate that acute exercise selectively affects N2-indexed functions—consistent with modulation of go-related preparation/selection processes in go-related response preparation/selection—while N2 measures of stop-related conflict monitoring remain largely unchanged within the sensitivity of our measures.

The P3 component is also elicited by go stimuli and is thought to reflect the completion of stimulus evaluation, monitoring of response outcomes, and the allocation of attentional resources to decision-making processes (Polich, 2007). The P3 is generally elicited more robustly by infrequent stimuli. Within the SST, Jia et al. (2021) manipulated the probability of stop-signal occurrence and reported that go-P3 amplitude decreased significantly as the stop-signal probability increased. In the present study, for goCor, although a significant main effect of condition on P3 amplitude was observed, the effect size was small and no pairwise differences survived *post hoc* correction, indicating that any practical influence on cognitive processes indexed by the go-P3 was limited.

Regarding the functional meaning of P3 in the SST more broadly, Huster et al. (2020) demonstrated that although P3 latency correlates with the behavioral index of stopping timing (SSRT), P3 amplitude does not show a consistent association with SSRT, suggesting that P3 may primarily reflect broader cognitive processes such as performance monitoring and behavioral adaptation rather than inhibition per se. This interpretation is further supported by evidence that P3 amplitude reflects context updating and attentional resource allocation, varies with task context (e.g., stop-signal probability) and is sensitive to SSD and outcome factors when contrasting successful versus unsuccessful stops; moreover, model-based accounts indicate that trigger failures can substantially influence ERP–behavior associations—together arguing against treating P3 amplitude as a specific marker of inhibition (Kok et al., 2004; Ramautar et al., 2004; Polich, 2007; Skippen et al., 2020). Taken together, these findings suggest that stop-P3 amplitude is better interpreted as reflecting the amount of higher-order cognitive control that accompanies inhibition—such as the allocation of attention to unexpected events and contextual updating—rather than a direct index of the success or strength of inhibition itself. In earlier work examining the impact of acute exercise, Chu et al. (2015) reported increased stop-P3 amplitudes in successful stop trials after acute exercise, suggesting the possibility that P3 modulation induced by acute exercise may emerge during the recovery period. In the present study, however, SStop-P3 amplitude showed no significant effects, neither during nor after exercise. While we did find that UStop-P3 amplitudes were larger during recovery_1 compared to the control period, the effect size was small. Overall, this pattern suggests a small increase limited to the recovery phase with restricted robustness. It should also be noted that stop-P3 amplitude in the SST can vary substantially with task manipulations, such as stop-signal probability or trial sequence (runs of consecutive go trials) (Ramautar et al., 2004; Zamorano et al., 2014), whereas prolonged monotonous tasks inducing mental fatigue have been associated with decreases in no-go-P3 in go/no-go tasks (Guo et al., 2018). In the present study, task parameters were held constant across conditions, suggesting that the modest recovery phase increase in stop-P3 amplitude likely reflects, at least in part, the acute exercise manipulation rather than variations in task context. Overall, our results indicate that the practical impact of acute exercise on performance monitoring and behavioral adaptation is likely modest, in line with the generally small effects of acute aerobic exercise on cognitive performance (Ishihara et al., 2021). Nevertheless, any mechanistic interpretation in terms of catecholaminergic arousal or hemodynamic changes should be considered speculative in the absence of direct physiological measures. Future work employing explicit tests of block/time factors will be useful to clarify the temporal profile and robustness of these effects.

### 4.3 Comparison with previous studies

With respect to the effects of acute exercise on cognitive function, transient improvements are most often observed at moderate-intensity exercise, and this pattern is supported by meta-analytic evidence (Chang et al., 2012). In contrast, findings regarding high-intensity exercise remain equivocal, with studies reporting both performance impairments (Smith et al., 2016) and performance facilitation (Huertas et al., 2011). In interpreting the present ‘high-intensity’ condition, it should be noted that the achieved intensity averaged approximately 65% HRR rather than the targeted 75% HRR, reflecting pragmatic workload adjustments within the ACSM-defined vigorous-intensity range to preserve EEG recording quality during cycling. Accordingly, conclusions about intensity–dose relationships at the upper end should be drawn cautiously; future studies incorporating additional physiological anchors (e.g., perceived exertion and/or hemodynamic measures) and refined intensity-control procedures will help better characterize the relationship between achieved intensity and neurocognitive outcomes. The present time course—behavioral facilitation primarily during exercise with attenuation in recovery—fits within this mixed literature and underscores the importance of assessment timing relative to exercise.

Using the SST, Joyce et al. (2009) showed that during ~30 min of moderate-intensity cycling, both goRT and SSRT were shortened, and that goRT and SSRT reductions persisted at 0–22 min and 30–52 min post-exercise. In contrast, in the present study, RT shortening was largely confined to the exercise period, and persistence into recovery was not evident. Differences between the two studies may reflect exercise-intensity settings (e.g., ~40% maximal aerobic power), the post-exercise observation windows (immediately post-exercise compared to 30–52 min after exercise), as well as SST parameters and sample size. In any case, exercise-induced facilitation is consistent across studies, whereas post-exercise persistence appears to vary, underscoring the need to identify further moderators of recovery-phase effects (e.g., exercise intensity, assessment timing, individual differences).

In addition, during moderate-intensity aerobic exercise, go/no-go RTs have repeatedly been shown to be shorter than at rest (Ando et al., 2013; Komiyama et al., 2016). These studies align with the present behavioral finding of faster response execution during exercise, whereas post-exercise effects again remain mixed across studies. Accordingly, the time course suggested here—transient facilitation during exercise with attenuation in recovery—is not at odds with the broader SST and go/no-go studies.

Systematic reviews and meta-analyses of exercise EEG further suggest that ERP modulations are not uniform across components, with P3 amplitude changes reported more consistently than N2 changes, whereas N2 findings appear more heterogeneous (e.g., Kao et al., 2025; Cai et al., 2025). Against this background, the present ERP pattern—robust modulation of goCor-N2 during exercise and only modest recovery-phase modulation of UStop-P3—supports the view that acute exercise effects may be selective across processing stages and time phases.

The novelty of the present study lies in extending measurement timing and adding ERP indices that together suggest a phase offset between time phases (control–exercise–recovery) and processing stages (preparation/selection vs. evaluation/monitoring). Specifically, during exercise, a reduction in goCor-N2 amplitude co-occurred with faster response execution. Subsequently, a small increase in UStop-P3 was observed early in recovery, suggesting a possible transient fluctuation in evaluation/monitoring processes after exertion. While the recovery-phase P3 effect was limited in magnitude and spatial robustness, the overall pattern is consistent with the idea that acute exercise can modulate different processing stages at different time points relative to exertion, with limited and variable carryover into recovery.

## 5 Conclusion

Overall, acute aerobic exercise was associated with a transient facilitation of response speed and stopping efficiency during exercise, with little evidence of persistence into the short recovery windows examined here. At the electrophysiological level, effects were selective, with a robust modulation of go-related N2 during exercise and at most a modest recovery-phase modulation of UStop-P3. Practically, these results suggest that any cognitive benefit of a single exercise bout may be time-locked to the period of exertion, underscoring the importance of assessment timing in acute-exercise interventions.

## Data Availability

The raw data supporting the conclusions of this article will be made available by the authors, without undue reservation.
