# Supplementary material for: Transient and selective effects of acute exercise intensity on response inhibition: an EEG study
Source: Front Hum Neurosci. 2026 Feb 19;20:1706674. doi: 10.3389/fnhum.2026.1706674 (PMC12960493; doi:10.3389/fnhum.2026.1706674)
Supplement: Supplementary file 1 [file Data_Sheet_1.PDF]

Supplementary Material

Supplementary Table S1. Participant disposition and exclusion reasons by exercise-intensity group.

	low	moderate	high	total
Participants tested	10	12	11	33
Excluded (total)	4	4	4	12
Excluded due to poor SST performance	2	3	2	7
Excluded due to substantial movement-related EEG artifacts	1	0	0	1
Excluded due to both SST performance and EEG artifacts	0	1	2	3
Excluded due to technical issue	1	0	0	1
Included in final analyses	6	8	7	21

Participants tested indicates the number of participants who completed the experiment. Participants meeting multiple exclusion criteria were categorized under both SST performance and EEG artifacts. Technical issue refers to missing or incomplete event markers required for epoching and ERP computation. SST, stop-signal task. EEG, electroencephalography.

Supplementary Table S2. Number of retained and rejected epochs by condition, trial type, and exercise-intensity group.

	Condition	low			moderate			high		
		Retained	Rejected	Retention rate (%)	Retained	Rejected	Retention rate (%)	Retained	Rejected	Retention rate (%)
goCor	control	66.7 (1.8)	1.7 (1.2)	97.6 (1.8)	66.4 (1.2)	1.2 (0.9)	98.2 (1.3)	65.3 (1.3)	1.9 (1.5)	97.2 (2.2)
	exercise	64.5 (2.3)	2.0 (1.8)	97.0 (2.7)	66.2 (1.8)	1.6 (1.1)	97.6 (1.6)	66.7 (2.0)	1.9 (1.3)	97.3 (2.0)
	recovery_1	67.3 (0.8)	0.8 (0.8)	98.8 (1.1)	65.8 (3.6)	2.9 (3.2)	95.8 (4.8)	65.6 (2.3)	2.4 (1.6)	96.4 (2.4)
	recovery_2	66.7 (1.6)	1.0 (1.1)	98.5 (1.6)	66.6 (1.4)	1.5 (0.9)	97.8 (1.3)	63.9 (4.5)	3.6 (2.1)	94.6 (3.6)
SStop	control	15.0 (2.7)	0.0 (0.0)	100.0 (0.0)	13.9 (1.4)	0.0 (0.0)	100.0 (0.0)	14.9 (0.7)	0.0 (0.0)	100.0 (0.0)
	exercise	13.5 (3.1)	0.0 (0.0)	100.0 (0.0)	13.8 (0.9)	0.0 (0.0)	100.0 (0.0)	13.6 (1.8)	0.0 (0.0)	100.0 (0.0)
	recovery_1	14.3 (2.5)	0.2 (0.4)	99.2 (2.0)	13.8 (1.3)	0.0 (0.0)	100.0 (0.0)	14.7 (1.8)	0.0 (0.0)	100.0 (0.0)
	recovery_2	14.0 (1.8)	0.0 (0.0)	100.0 (0.0)	13.9 (0.6)	0.0 (0.0)	100.0 (0.0)	15.6 (1.8)	0.0 (0.0)	100.0 (0.0)
UStop	control	14.3 (2.7)	0.0 (0.0)	100.0 (0.0)	15.8 (1.2)	0.0 (0.0)	100.0 (0.0)	14.9 (1.1)	0.0 (0.0)	100.0 (0.0)
	exercise	16.3 (2.9)	0.0 (0.0)	100.0 (0.0)	15.4 (1.1)	0.0 (0.0)	100.0 (0.0)	15.6 (1.5)	0.0 (0.0)	100.0 (0.0)
	recovery_1	15.3 (2.7)	0.0 (0.0)	100.0 (0.0)	15.4 (1.5)	0.0 (0.0)	100.0 (0.0)	15.0 (1.7)	0.0 (0.0)	100.0 (0.0)
	recovery_2	15.8 (1.9)	0.0 (0.0)	100.0 (0.0)	15.8 (1.0)	0.0 (0.0)	100.0 (0.0)	13.7 (2.0)	0.0 (0.0)	100.0 (0.0)

Values are shown as mean (SD) across participants. "Retained" indicates the number of epochs included in ERP averaging after artifact rejection; "Rejected" indicates epochs removed by artifact rejection. Retention rate (%) = retained / (retained + rejected) × 100.

Supplementary Table S3. ICA component rejection summary by exercise-intensity group.

	low	moderate	high
Removed ICs (n)	11.5 (2.4)	13.5 (2.7)	14.4 (3.8)
ICA component removal rate (%)	38.3 (8.1)	45.0 (9.1)	48.4 (12.9)

Values are reported as mean plus or minus SD across participants. Rejected ICs indicates the number of independent components removed during ICA-based artifact correction. ICA rejection rate indicates the percentage of removed components relative to the total number of components in the ICA decomposition. ICA, independent component analysis. IC, independent component.

Supplementary Table S4. Peak latencies for P2, N2, and P3 by condition, trial type, and exercise-intensity group.

P2 (Fz)	Condition	low		moderate		high	
		mean	(SD)	mean	(SD)	mean	(SD)
goCor	control	178.33	(36.74)	208.25	(29.01)	198.00	(28.55)
	exercise	189.00	(32.72)	201.75	(34.23)	190.29	(42.38)
	recovery_1	177.67	(37.39)	179.75	(50.60)	194.57	(29.10)
	recovery_2	189.67	(21.40)	163.75	(49.34)	195.43	(23.80)
SStop	control	205.33	(27.54)	169.00	(45.55)	165.14	(51.43)
	exercise	151.67	(43.52)	179.50	(30.61)	177.71	(58.08)
	recovery_1	172.33	(43.79)	176.50	(41.40)	170.86	(37.75)
	recovery_2	193.33	(25.32)	167.75	(43.96)	197.71	(14.40)
UStop	control	216.00	(17.55)	195.00	(40.04)	205.43	(18.78)
	exercise	169.00	(47.91)	194.50	(20.54)	170.57	(36.09)
	recovery_1	189.33	(40.75)	182.25	(50.68)	186.00	(38.65)
	recovery_2	196.33	(18.53)	197.50	(17.40)	181.43	(41.57)
**N2 (Cz)**	**Condition**	**low**		**moderate**		**high**	
		**mean**	**(SD)**	**mean**	**(SD)**	**mean**	**(SD)**
goCor	control	250.33	(49.85)	236.25	(68.24)	244.00	(58.46)
	exercise	217.67	(40.09)	243.50	(57.41)	258.00	(53.12)
	recovery_1	224.00	(65.31)	243.50	(57.38)	239.14	(58.65)
	recovery_2	244.00	(48.65)	260.25	(44.68)	278.86	(16.93)
SStop	control	209.33	(41.71)	172.50	(34.88)	188.00	(43.95)
	exercise	204.33	(47.87)	169.75	(32.50)	204.57	(52.72)
	recovery_1	220.33	(55.61)	190.75	(51.82)	184.57	(41.63)
	recovery_2	213.67	(39.25)	183.75	(30.11)	220.29	(48.48)
UStop	control	193.00	(37.55)	196.75	(56.70)	236.29	(57.32)
	exercise	242.33	(53.21)	200.50	(47.37)	255.71	(50.70)
	recovery_1	217.00	(52.43)	213.25	(61.36)	261.71	(22.36)
	recovery_2	258.67	(32.90)	221.50	(56.84)	232.86	(38.22)
**P3 (Cz)**	**Condition**	**low**		**moderate**		**high**	

		mean	(SD)	mean	(SD)	mean	(SD)
goCor	control	386.67	(75.14)	450.50	(55.85)	450.57	(60.28)
	exercise	457.00	(63.73)	466.25	(37.79)	388.57	(81.68)
	recovery_1	402.67	(98.73)	456.50	(51.50)	436.00	(62.09)
	recovery_2	397.00	(76.61)	457.50	(37.56)	420.00	(66.74)
SStop	control	326.33	(35.93)	318.00	(43.84)	322.57	(48.90)
	exercise	327.67	(34.38)	323.00	(70.07)	331.43	(49.75)
	recovery_1	326.67	(43.48)	339.00	(73.61)	338.29	(32.85)
	recovery_2	341.67	(47.01)	366.25	(69.33)	343.43	(36.98)
UStop	control	385.67	(37.07)	394.25	(41.35)	388.00	(32.34)
	exercise	368.33	(33.61)	353.00	(49.67)	373.43	(16.27)
	recovery_1	359.67	(44.91)	385.25	(31.32)	368.29	(16.26)
	recovery_2	353.67	(27.46)	373.25	(30.69)	353.43	(34.95)

Values are reported as mean plus or minus SD in milliseconds. Latencies were extracted at the electrode used for the corresponding amplitude analysis, P2 at Fz and N2 and P3 at Cz. Peak latency was defined as the time point of the maximum positive peak for P2 and P3 and the maximum negative peak for N2 within the pre-defined time window used for the mean peak amplitude. For goCor, epochs were time-locked to the go-signal onset. For SStop and UStop, epochs were time-locked to the stop-signal onset. Latency values are provided for descriptive purposes only and were not subjected to inferential statistical testing.
